# The influence of HK2 blood group antigen on human B cell activation for ABOi-KT conditions

**DOI:** 10.1186/s12865-017-0233-9

**Published:** 2017-12-16

**Authors:** Jingsong Cao, Luogen Liu, Yunsheng Zhang, Jianhua Xiao, Yi Wang

**Affiliations:** 1 Institute of Pathogenic Biology, Medical College, Hunan Provincial Key Laboratory for Special Pathogens Prevention and Control; Hunan Province Cooperative Innovation Center for Molecular Target New Drug Study, University of South China, Hengyang, Hunan 421001 China; 2 Clinical research center, Institute of Pathogenic Biology, Medical College, The Second Affiliated Hospital, University of South China, Hengyang, Hunan 421001 China; 3 Urinary surgery, The Second Affiliated Hospital, University of South China, Hengyang, Hunan 421001 China

**Keywords:** Blood group B antigen, Blood group B antibody, HK2, B cells activation, ABOi-KT

## Abstract

**Background:**

It is well known that ABO blood group system incompatible kidney transplantation (ABOi-KT) is an effective strategy for end-stage renal disease. The main barrier for ABOi-KT is how to keep host B cell activation and blood group antibody titer in low levels. Moreover, the mechanism of B cell activation induced by blood group antigen was unclear in ABOi-KT.

**Results:**

In this study, HK2 cells were identified to express blood group B antigen when cocultured with lymphocytes of blood group A. Optical microscope observation demonstrated that HK2 cells in coculture group gradually decreased. Furthermore, flow cytometer assay identified that T cell phenotypes (CD3^+^, CD3^+^CD4^+^ and CD3^+^CD8^+^) had no significant change and B cell phenotypes (CD19^+^ and CD138^+^) were all significantly enhanced (3.07 and 3.02 folds) at day 4. In addition, immunoturbidimetry analysis demonstrated that blood group B antibody was significantly increased to 2.35 fold at day 4, IgG was significantly increased to 3.60 and 2.81 folds at days 4 and 8 respectively, while IgM had no significant change at the measured time points.

**Conclusions:**

Taken together, B cells were activated and secreted blood group B antibody after treatment with HK2 expressing blood group B antigen. The results of this study maybe useful for further determination of the mechanism of B cell activation after ABO incompatible kidney endothelial cells stimulation.

**Electronic supplementary material:**

The online version of this article (10.1186/s12865-017-0233-9) contains supplementary material, which is available to authorized users.

## Background

ABOi-KT is an effective replacement therapy for end-stage kidney disease [1–3], in which the key for graft survival is to eliminate the host blood group antibodies prestored in peripheral blood of recipients [4, 5]. However, the allograft in part of ABOi-KT recipients survived without rejection when the blood group antibody titer was gradually increased to the preoperative level [6].

Some researchers considered the allograft survival was related to immune tolerance mediated by antibodies [7, 8]. Urschel et al. showed CD21-expressing B cells were related to ABO tolerance [9]. Chesneau et al. reported a unique B cell in vitro differentiation profile that played an important role in tolerant kidney transplant patients [10], especially the isotype of immunoglobulin (Ig) on the surface of B cells switch from IgM to IgG [11, 12]. Methot et al. [13] noted that B cell differentiation resulted in antibody diversification, which impacted the antibodies activity for binding to Fc receptors and activation of the complement system [8]. However, the mechanism of B cells activation in ABOi-KT was unclear.

In this study, HK2 cells were identified to express blood group B antigen. After coculture with lymphocytes isolated from blood group A health donors, the HK2 cells were observed by optical microscopy. Of these, the lymphocytes phenotype, such as CD3^+^, CD3^+^CD4^+^, CD3^+^CD8^+^, CD19^+^ and CD138^+^, were analyzed by flow cytometry. Furthermore, the blood group B antibody, IgG and IgM were detected by immunoturbidimetry assay. These results will be beneficial for further exploration of the mechanism of B cells activation after ABO incompatible kidney endothelial cells stimulation.

## Methods

HK2 cell line was purchased from the Advanced Research Center of Central South University. The peripheral blood was donated from volunteers after informed consent, and subsequently approved by the Animal Welfare and Research Ethics Committee of the Institute of University of South China.

The specificity glycosyl of blood group B antigen was synthesized and coupled to keyhole limpet hemocyanin (KLH-B) at Alberta Innovates Technology Futures. The KLH-B was dissolved in phosphate buffer solution (PBS, 0.01 mol/L, pH 7.4) to 0.001 mg/ml.

### Cell culture

Lymphocytes were separated from blood group A donors and cultured as Cao et al. [14] reported with some modification. Peripheral blood at 2 ml was mixed with 0.9% physiological saline (*V*:*V* = 1:1) for ficoll gradient separation (LymphoPrep). After centrifugation at 1800 revolutions/min for 20 min, the lymphocytes layer was collected and rinsed 2 times with 0.9% physiological saline at 1500 revolutions/min for 7 min. Then the cells were resuspended with 1640 medium (Thermo Fisher Scientific) and 15% fetal calf serum (FCS, Thermo Fisher Scientific) to 2 × 10^6^ cells/ml.

The HK2 cells in dish culture were processed by 3 ml 0.25% trypsin (GE Healthcare Life Sciences) at room temperature for 2 minnutes, then 3 ml 1640 medium with 15% FCS added, and centrifuged at 800 revolutions/min for 10 min. Afterwards was rinsed 2 times with 0.9% physiological saline at 800 revolutions/min for 10 min, the precipitate was resuspended with 1640 medium and 15% FCS to 2 × 10^6^ cells/ml.

Then, leukocytes and HK2 cells were divided into three groups, HK2 group was added 0.5 ml HK2 cells suspension and 0.5 ml 1640 medium with 15% FCS, PB group was added 0.5 ml lymphocytes suspension and 0.5 ml 1640 medium with 15% FCS, coculture group was added 0.5 ml HK2 cells suspension and 0.5 ml lymphocytes suspension. The three groups were all cultured in 24-well plates at 37 °C, 5% CO_2_, and added 0.1 ml fresh medium to every group at day 4. The experiment was repeated for 3 times.

### Immunohistochemistry assay

The process of immunohistochemistry was built as Kounelis [15] reported with some modification. HK2 cells were cultured with 1640 medium and 15% FCS at 37 °C, 5% CO_2_. After rinsed with PBS (0.01 mol/L, pH 7.2) for 2 times, the carry sheet glasses of HK2 cells were incubated with FCS at 37 °C for 20 min. Then the carry sheet glasses were divided into three groups, one group was incubated with 1 ml PBS at 37 °C for 1 h, the other two groups were respectively incubated with mouse monoclonal to anti-blood group A antibody (1: 100, Albanian Broadband Communication) and mouse monoclonal to anti-blood group B antibody (1: 100, Albanian Broadband Communication) at 37 °C for 1 h. After rinsed 3 times with PBS for 2 min, the three groups were all incubated with goat anti-mouse IgM-HRP (1: 500, ThermoFisher Scientific) at 37 °C for 20 min, followed rinsed 3 times with PBS for 2 min. Finally, the carry sheet glasses were rinsed 3 times with PBS for 2 min and analyzed with DAB Kit (BOSTER) detection method. The experiment was repeated for 3 times.

### Optical microscope observation

At days 2, 4, 8, the lymphocytes were collected for further research. HK2 cells were observed by optical microscopy after two PBS rinses. The experiment was repeated for 3 times.

### Flow cytometer assay

At days 2, 4, 8, the lymphocytes suspension were collected by concentrated at 1000 revolutions/min for 10 min, then the precipitate was resuspended with 1 ml 0.9% physiological saline, after centrifuged at 1000 revolutions/min for 10 min, the precipitate was resuspended with 150 μl 0.9% physiological saline, and divided into three groups. One group as isotype control was added FITC mouse IgG2α (5 μl, BD), PE mouse IgG1 (5 μl, BD) and PerCP-CyTM 5.5 mouse IgG1 (1 μl, BD), the experiment group was divided into two group and, respectively, added FITC mouse anti-human CD3 (5 μl, BD), PE mouse anti-human CD4 (5 μl, BD), PerCP-CyTM5.5 mouse anti-human CD8 (1 μl, BD) and FITC mouse anti-human CD19 (5 μl, BD), PE mouse anti-human CD138 (5 μl, BD). The three groups were all incubated at room temperature for 15 min, then resuspended with 1 ml 0.9% physiological saline and centrifuged at 1000 revolutions/min for 10 min. Finally, the precipitate was resuspended with 0.2 ml 0.9% physiological saline and prepared to analysis using BD FACSCanto II. The experiment was repeated for 3 times.

### Detection of the concentration of mouse monoclonal to anti-blood group B antibody in reagent

After polyacrylamide gel electrophoresis (PAGE, 8% separation gel) of mouse monoclonal to anti-blood group B antibody reagent, the gel was transferred to a polyvinylidene fluoride (PVDF) membrane for 90 minnutes at 300 mA in transfer buffer (0.025 mol/L Tris, 0.1 mol/L glycine, and 20% methanol), and the membrane was blocked for 1 h in tween tris buffer solution (TTBS, 0.05% Tween-20, 0.02 mol/L Tris, 0.15 mol/L NaCl, pH 7.4) containing 5% skim milk at room temperate. After rinsed 3 times with tris buffer solution (TBS) for 5 min, the membrane was incubated with goat anti-mouse IgM-HRP (1: 2000) at room temperate for 40 min. Then the membrane was rinsed 2 times with TBS for 15 min and analyzed with ECL Plus (solarbio) chemiluminescence detection method.

In addition, the concentration of mouse monoclonal to anti-blood group B antibody was calculated by protein-gray value curve of bovine serum albumin (BSA). The experiment was repeated for 3 times.

### Immunoturbidimetry assay

At days 2, 4, 8, the supernate was collected. Experiment group was a mixture of the supernate, buffer solution (0.01 mol/L, pH 7.4 PBS, 0.15 mol/L NaCl, 40 g/L PEG-8000) and KLH-B (*V*: *V*: *V* = 1: 94: 5). positive control group was a mixture of mouse monoclonal to anti-blood group B antibody, buffer solution and KLH-B (*V*: *V*: *V* = 1: 94: 5). Blank group was a mixture of buffer solution and KLH-B (*V*: *V* = 95: 5). All groups were incubated at room temperature for 5 min, and then detected the absorb value at λ_340nm_.

Meanwhile, The IgM and IgG in supernate were respectively analyzed by N Antiserum to Human IgM reagent (Siemens) and N Antiserum to Human IgG reagent (Siemens). The experiments were all repeated for 3 times.

## Results

### Identification of blood group antigen type of HK2 cells

**Fig. 1:**
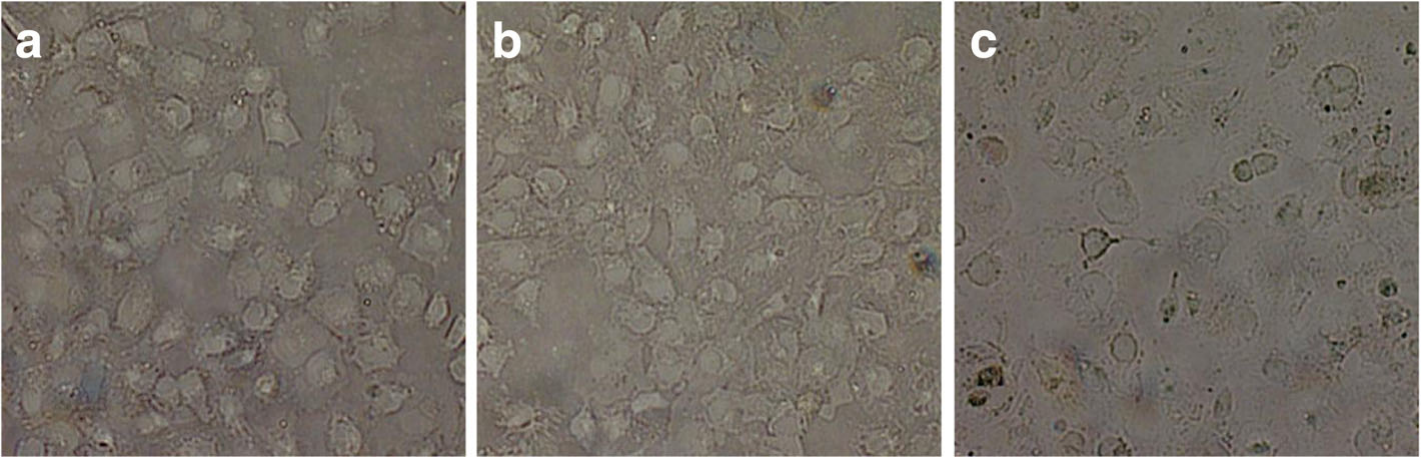
Blood group antigen identified by immunohistochemistry. **a** PB group, **b** Blood group A antigen analysis, **c** Blood group B antigen analysis. All groups were repeated for 3 times

To research the change of B cell activation by blood group antigens of kidney endotheliocytes stimulated in vitro, the blood group antigen type of the HK2 cell line was analyzed by immunohistochemistry (Fig. 1). As for results, none of the imaged HK2 cells were stained after incubation with PBS and mouse monoclonal to anti-blood group A antibody (Fig. 1 a, b). However, the HK2 cells could specifically link with mouse monoclonal to anti-blood group B antibody (Fig. 1c).

### Observation of HK2 cells proliferation

**Fig. 2:**
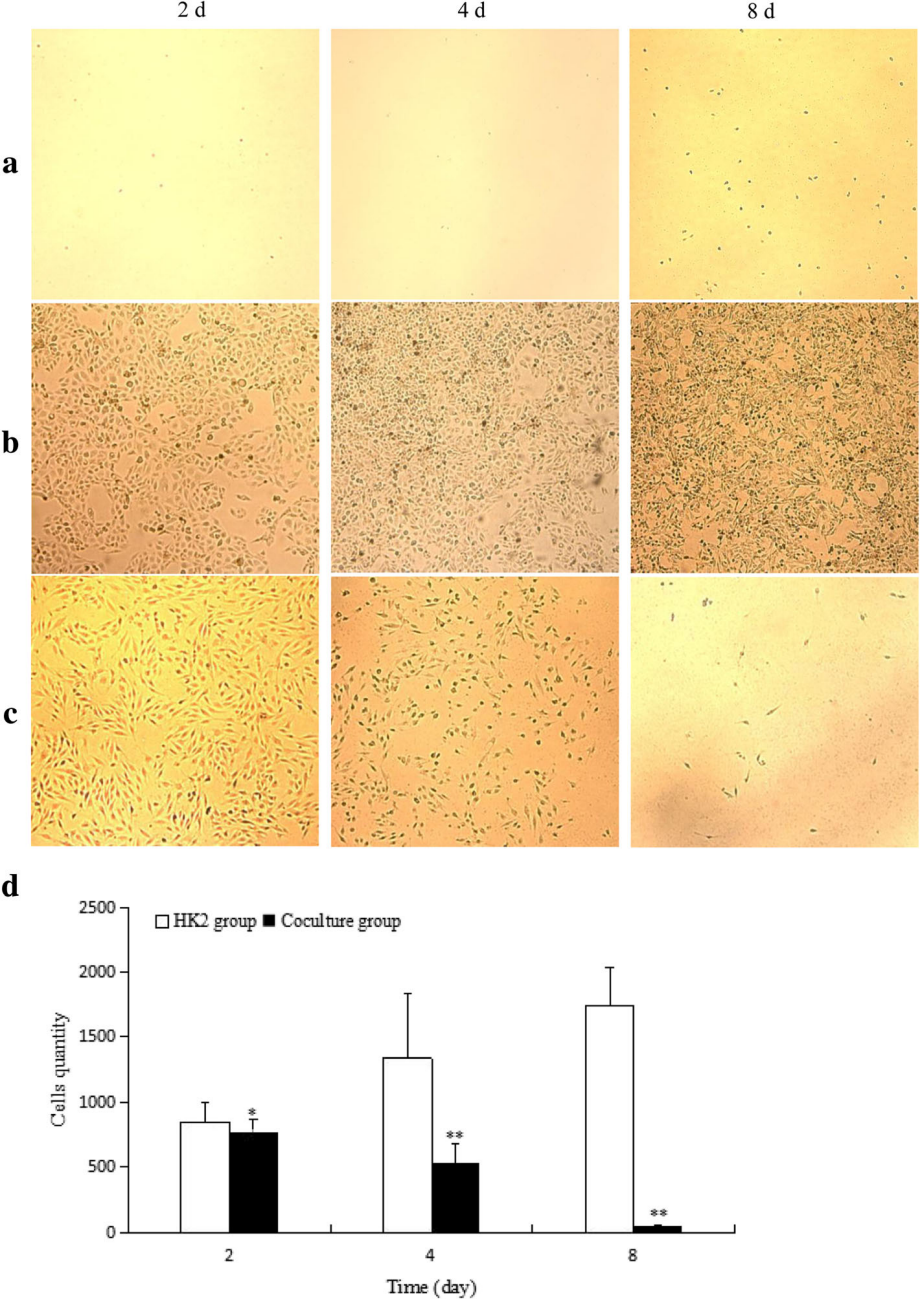
Optical microscope analyzed for HK2 cells. **a** PB group, **b** HK2 group, **c** Coculture group. **d** the analysis of histogram for HK2 cell quantity. Bars represent mean ± standard deviation (*n* = 3). Significant differences between the PB and the coculture groups were indicated with one (*p* < 0.05) or two (*p* < 0.01) asterisks

Following the results of immunohistochemistry, lymphocytes of blood group A were collected and cultured with HK2 cells. At days 2, 4, 8, HK2 cells were observed by optical microscopy (Fig. 2). Compared with PB (Fig. 2a) and HK2 groups (Fig. 2b, d), the proliferative activity of HK2 cells in coculture group was decreased and a small number of HK2 cells survived at day 8 (Fig. 2c, d).

### Analysis of lymphocytes phenotype

**Fig. 3:**
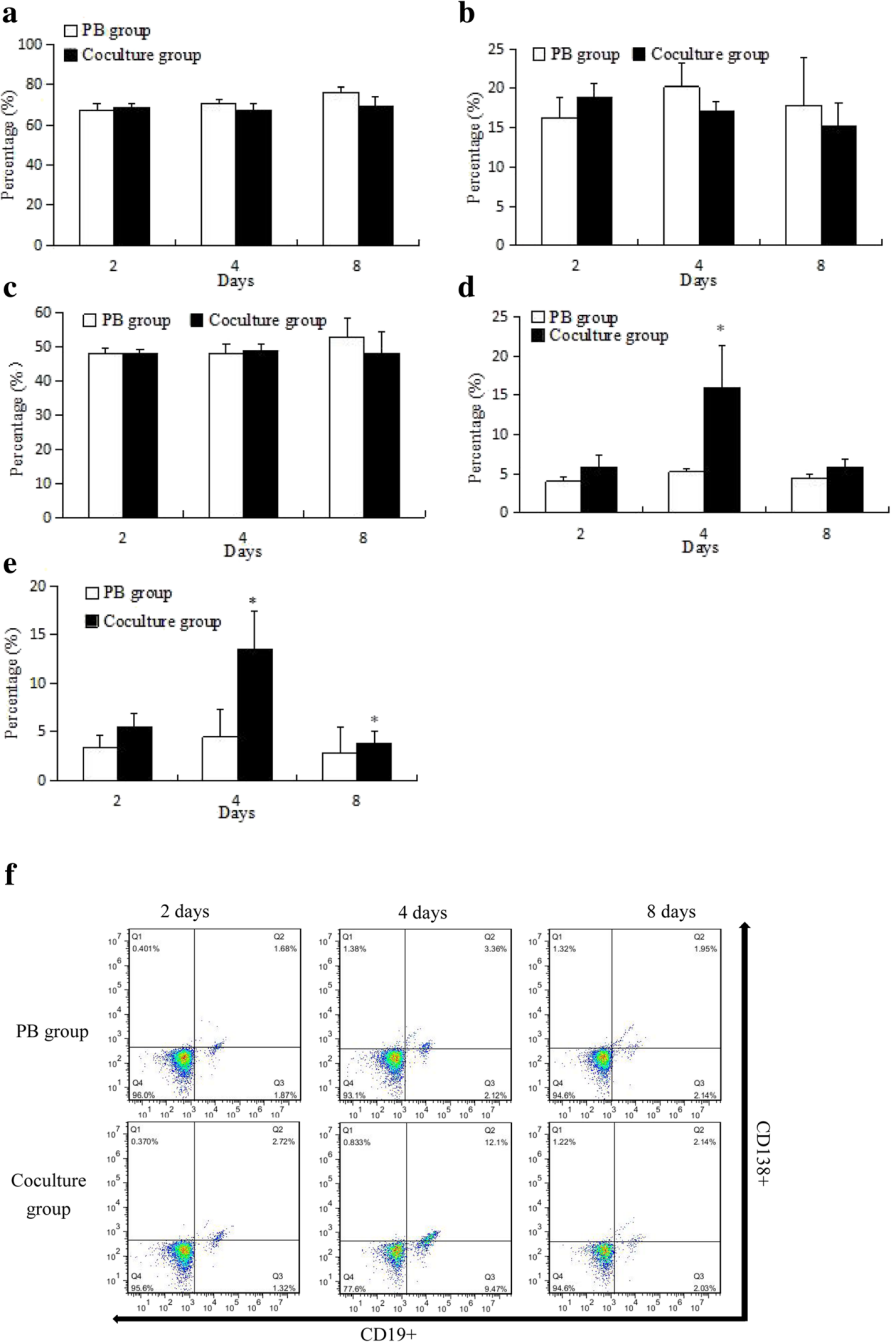
The T cells and B cells subsets analyzed by flow cytometry. **a** CD3^+^ phenotype T cells, **b** CD3^+^CD8^+^ phenotype T cells, **c** CD3^+^CD4^+^ phenotype T cells, **d** CD19^+^ phenotype B cells, **e** CD138^+^ phenotype B cells. **f** The flow cytometry scatter plot of CD19^+^ and CD138^+^ phenotype B cells. Bars represent mean ± standard deviation (n = 3). Significant differences between the PB and the coculture groups were indicated with one (p < 0.05) asterisks

To demonstrate the lymphocyte subtypes influencing HK2 cells proliferative activity, the T cells phenotype (CD3^+^, CD3^+^CD4^+^ and CD3^+^CD8^+^) and B cells phenotype (CD19^+^ and CD138^+^) were analyzed by flow cytometer. As Fig. 3 appeared, compared with PB group, T cell phenotypes (Fig. 3a–c) had no significant change, and CD19^+^ (Fig. 3d, f) B cells were significantly increased to 3.07 fold at day 4, CD138^+^ (Fig. 3e, f) B cells were significantly increased to 3.02 and 1.36 folds at days 4 and 8 respectively.

### Determination of the anti-blood group B mouse monoclonal antibody concentration

**Fig. 4:**
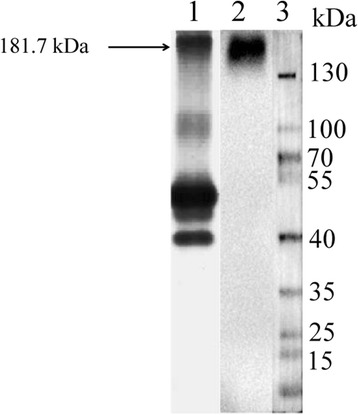
The analysis of western-blotting for mouse monoclonal to anti-blood group B antibody in reagent. Lane 1. PAGE assay, lane 2. Western-blotting assay, lane 3. protein mark

For further exploration of the variation of blood group B antibody secreted by B cells, the concentration of mouse monoclonal to anti-blood group B antibody in reagent was analyzed. As Fig. 4 showed, in the reagent, only the protein with a molecular weight about 181.7 kDa was specifically combined with goat anti mouse IgM-HRP. After comparison with the standard curve of BSA (Additional file 1: Figure S1), the mouse monoclonal to anti-blood group B antibody concentration was identified as 0.57 mg/ml.

### Detection of the concentration of blood group antibody, IgG and IgM

**Fig. 5:**
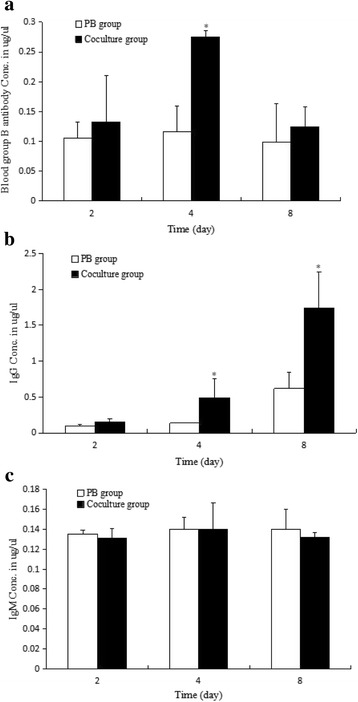
Immunoturbidimetry analysis for blood group B antibody, IgG and IgM. **a** Blood group B antibody concentration assay; **b** IgG concentration assay; **c** IgM concentration assay. Bars represent mean ± standard deviation (n = 3). Significant differences between the PB and the coculture group were indicated with one (p < 0.05) or two (p < 0.01) asterisks. Conc.: concentration

Based on these results, the concentration of blood group B antibody, IgG and IgM in supernate was analyzed by immunoturbidimetry assay. As Fig. 5 showed, blood group B antibody was significantly increased to 2.35 fold at day 4 (Fig. 5a), IgG was significantly increased to 3.60 and 2.81 folds at days 4 and 8 respectively (Fig. 5b), and IgM had no significant change between PB and coculture groups (Fig. 5c).

## Discussion

ABOi-KT is an effective and important strategy to resolve the problem of kidney shortage [16–21], and the allograft in a small cohort of recipients aided survival because of immune tolerance mediated by B cells activation [22, 23]. In this study, HK2 was verified to express blood group B antigen (Fig. 1) when cocultured with lymphocyte of blood group A.

Optical microscope observation shown that the proliferative activity of HK2 cells in coculture group decreased and a small number of HK2 cells survived by day 8 (Fig. 2c, d). This phenomenon may be related to humoral immunity mediated by B cells or cell immunity mediated by T cells [24–26].

**Fig. 6:**
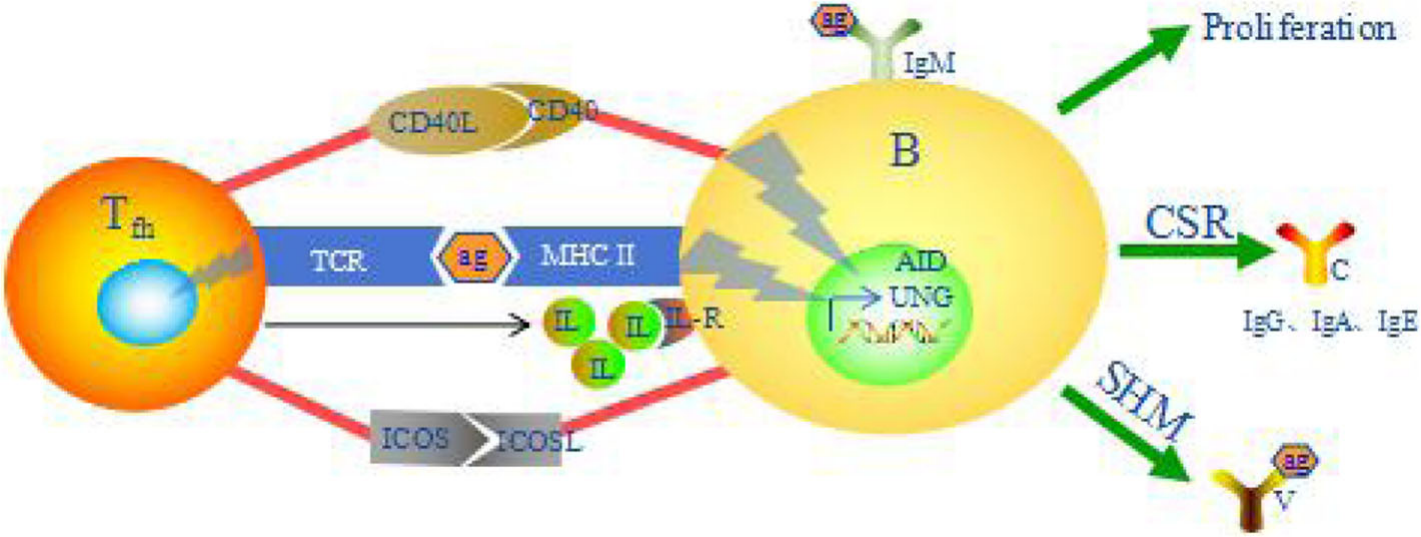
The activation of T follicular helper cells for B cells*. T_fh_: T follicular helper, TCR: T cell receptor, MHC II: major histocompatibility complex class II, ICOS: Inducible costimulator, ICOSL: ICOS ligand, CD40L: CD40 ligand, IL: interleukin, IL-R: interleukin receptor, CSR: class switch recombination, SHM: somatic hypermutation, AID: activation-induced cytidine deaminase, UNG: uracil-N-glycosylase. * The figure was drawn as Durandy er al [35] reported with some modification

In immunoreactions mediated by lymphocytes, CD3^+^CD4^+^ and CD3^+^CD8^+^ T cells play an important role as helper T cells [27] and cytotoxic T cells [28]. The CD3^+^CD4^+^ T cells, especially T follicular helper (Tfh) cells, secrete interleukin-21 [29] and express CD40 ligands and other inducible costimulator such as CD137 [30] on their surface [31], to promote B cells (CD19^+^) class-switch and their differentiation into plasma cells (CD138^+^) after antigen challenge [32–34] (Fig. 6) [35]. Thus, the T cells (CD3^+^, CD3^+^CD4^+^ and CD3^+^CD8^+^) and B cells (CD19^+^ and CD138^+^) subset were analyzed by flow cytometry. As a result, after stimulation with HK2, the T cells phenotype had no significant variation (Fig. 3a–c), CD19^+^ B cells (Fig. 3d, f) were significantly increased to 3.07 fold at day 4, and CD138^+^ B cells (Fig. 3e, f) were significantly increased to 3.02 and 1.36 folds at days 4 and 8 respectively. Conjecturally, the B cells activation was induced by HK2, and it may be an important factor for the decrease of HK2 cells proliferative activity.

In addition, for further quantitative analysis the variation of blood group B antibody concentration, the mouse monoclonal to anti-blood group B antibody in reagent was detected as Eppler A et al. [36] and Yawata K et al. [37] reported with some modification. As a result, the protein with a molecular weight about 181.7 kDa was identified as mouse anti-human blood group B antibody with concentration 0.57 mg/ml in the reagent (Fig. 4 and Additional file 1: Figure S1).

Based on these results, the blood group antibody in the supernate was analyzed by immunoturbidimetry. The results showed that blood group B antibody was significantly increased to 2.35 fold at day 4 (Fig. 5a), IgG was significantly increased to 3.60 and 2.81 fold at days 4, 8 (Fig. 5b), and IgM has no significant change at the detective point (Fig. 5c). This was similar to the report that immunogloblulin was class switched to IgG4 in warthin tumor and the serum IgG4 levels were increased [7]. Consequently, it is suggested the B cells were activated by HK2 cells expressing blood group B antigen and blood group B antibodies played a key role in HK2 cells apoptosis.

## Conclusions

Our researches demostrated that the B cells were activated and induced differentiation to plasmocytes secreted blood group B antibody after HK2 cells treatment. This will be beneficial for further exploration of the mechanism of B cells activation after ABOi HK2 cells stimulation.

## Additional file

Additional file 1: Figure S1. the establishment of protein-gray value curve of BSA. (A) PAGE assay for different concentration of BSA, lane 1. 0.5 μg BSA, lane 2. 1 μg BSA, lane 3. 2 μg BSA, lane 4. 4 μg BSA, lane 5. 8 μg BSA; (B) the relationship analysis of BSA gray value and protein. Bars represent mean ± standard deviation (*n* = 3). (PDF 611 kb)

## Abbreviations

ABOi-KT: ABO blood group system incompatible kidney transplantation
AID: Activation-induced cytidine deaminase
BSA: Bovine serum albumin
CD40L: CD40 ligand
Conc.: Concentration
CSR: Class switch recombination
FCS: Fetal calf serum
ICOS: Inducible costimulator
ICOSL: ICOS ligand
Ig: Immunoglobulin
IL: Interleukin
IL-R: Interleukin receptor
KLH: Keyhole limpet hemocyanin
MHC II: Major histocompatibility complex class II
PAGE: Polyacrylamide gel electrophoresis
PBS: Phosphate buffer solution
PVDF: Polyvinylidene fluoride
S.D.: Standard deviation
SHM: Somatic hypermutation
TBS: Tris buffer solution
TCR: T cell receptor
T_fh_: T follicular helper
TTBS: Tween tris buffer solution
UNG: uracil-N-glycosylase
