# Supplementary figures and images for: The influence of HK2 blood group antigen on human B cell activation for ABOi-KT conditions
Source: BMC Immunol. 2017 Dec 16;18:49. doi: 10.1186/s12865-017-0233-9 (PMC5732526; doi:10.1186/s12865-017-0233-9)

Figure S1

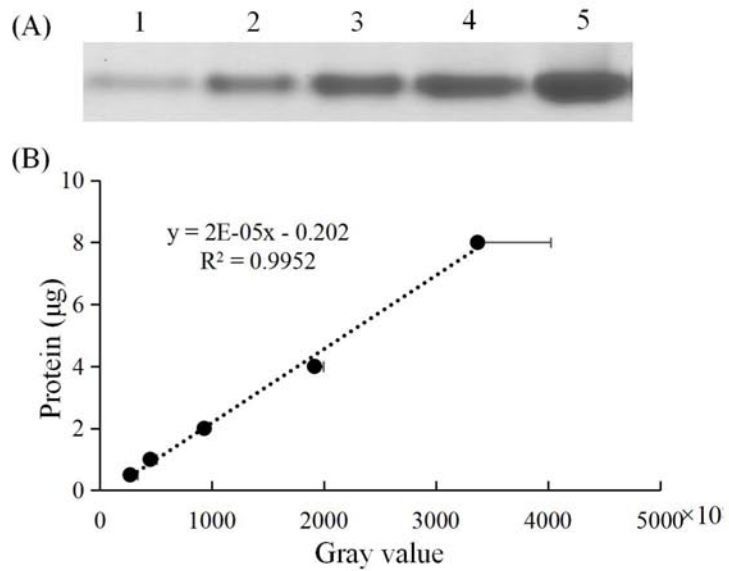

Supplement: Additional file 1: Figure S1. — the establishment of protein-gray value curve of BSA. (A) PAGE assay for different concentration of BSA, lane 1. 0.5 μg BSA, lane 2. 1 μg BSA, lane 3. 2 μg BSA, lane 4. 4 μg BSA, lane 5. 8 μg BSA; (B) the relationship analysis of BSA gray value and protein. Bars represent mean ± standard deviation (n = 3). (PDF 611 kb) [file 12865_2017_233_MOESM1_ESM.pdf]
